# Recurrent BMP4 variants in exon 4 cause non-HFE-associated hemochromatosis via the BMP/SMAD signaling pathway

**DOI:** 10.1186/s13023-024-03439-9

**Published:** 2024-11-19

**Authors:** Qin Ouyang, Yanmeng Li, Anjian Xu, Ning Zhang, Sisi Chen, Donghu Zhou, Bei Zhang, Xiaojuan Ou, Jidong Jia, Jian Huang, Wei Zhang

**Affiliations:** 1 Laboratory of Molecular Biology, Beijing Institute of Clinical Medicine, Beijing Friendship Hospital, Capital Medical University, Beijing, 100050 China; 2 Liver Research Center, Beijing Friendship Hospital, Capital Medical University, Beijing, 100050 China

**Keywords:** Hemochromatosis, *BMP4* p.H251Y variant, *BMP4* p.R269Q variant, Hepcidin, BMP/SMAD

## Abstract

**Background:**

Hereditary hemochromatosis (HH) is an iron overload disorder and can be caused by variants in non-*HFE* genes in Chinese patients. However, there is still a considerable proportion of patients suffering from unexplained iron overload. In our previous study, we had identified the p.R269Q variant in exon 4 of the Bone morphogenetic protein 4 (*BMP4*) gene in Chinese patients with unexplained primary iron overload by Whole Exome sequencing, and then the *BMP4* p.H251Y variant was identified by Sanger sequencing in a Chinese patient with secondary iron overload. Our study aimed to explore the pathogenicity and underlying mechanism of *BMP4* p.H251Y and *BMP4* p.R269Q variants in patients with iron overload.

**Methods:**

Sanger sequencing was conducted to identify the novel variants in the *BMP4* gene of patients with unexplained iron overload. MRI and liver biopsy were used to display iron overload in the liver of the patient harboring the *BMP4* p.H251Y variant. The BMP4 and hepcidin levels in BMP4 knockdown and BMP4 variant cells were examined by enzyme-linked immunosorbent assay. The effects of BMP4 p.H251Y and BMP4 p.R269Q variants on the hepcidin-regulation pathway were studied.

**Results:**

One of 54 HH patients (1.85%) harbored the *BMP4* p.R269Q variant. One of 148 patients (0.68%) with secondary hemochromatosis harbored the *BMP4* p.H251Y variant, and these two variants were not found in 100 Chinese general population. For the patient harboring the *BMP4* p.H251Y variant, abdominal MRI and Perl's staining of liver tissue displayed iron overload in the liver. Cells transfected with the BMP4 p.H251Y and p.R269Q variants showed down-regulation of hepcidin level and BMP/SMAD pathway compared with cells transfected with the wild-type BMP4 vector.

**Conclusion:**

The BMP4 p.H251Y and p.R269Q variants can downregulate hepcidin levels by inhibiting the BMP/SMAD axis, suggesting they may play pathogenic roles in iron overload.

**Supplementary Information:**

The online version contains supplementary material available at 10.1186/s13023-024-03439-9.

## Introduction

Hereditary hemochromatosis (HH) is a genetic iron overload disorder caused by hepcidin deficiency or a reduction in the hepcidin-ferroprotein combination, resulting in a variety of symptoms, such as hepatic dysfunction, cardiomyopathy, and diabetes [1]. The prevalence of genetic mutations that can cause HH differs among ethnic groups. These mutations occur in genes including homeostatic iron regulator (*HFE*), hemojuvelin (*HJV*), hepcidin (*HAMP*), transferrin receptor 2 (*TFR2*), and solute carrier family 40 member 1 (*SLC40A1*) [2]. Although European populations display a high frequency of the *HFE* p.C282Y mutation, most HH patients in Asia–Pacific countries are negative for *HFE* gene tests, making definitive diagnosis more difficult [3].

Previous case reports had identified several non-*HFE* variants, including *HAMP* p. R75X, *HJV* p.Y150C/p.V274M, and *TFR2* p.L490R, V561X in Japanese HH patients [4–6]; *HJV* p.Arg63Ter, p.Asp149ThrfsTer97 and *TFR2* p.Asp680Tyr, Trp781Ter, p.Gln672Ter in Spanish HH patients [7]; and *TFR2* p.G430R/p.Y320X, p.A356fs, p.I238M and p.G430R, *HJV* p.I287S and Y46X, and *SLC40A1* p.209L, IVS 3 + 10 del in Chinese HH patients [8–10]. Furthermore, our group had found certain variants in HH cases, including *TFR2* p.A302E and p.L745R, *HJV* p.E3D, p.H104R, and p.V274M, and *SLC40A1* p.Y333H [11, 12]. In addition, a series of novel pathogenic variants had also been identified that are involved in cellular iron homeostasis, such as *TMPRSS6* p.T331M, *DENND3* p.L708V, *SUGP2* p.R639Q, *UBE2O* p.K689R, and *PCSK7* p.V143F and p.R711W [11, 13, 14]. Our previous studies concluded that the HH gene mutation pattern in China may be characterized by mutations in non-*HFE* genes [11]. We also identified heterozygous p.R269Q and p.H251Y variants in exon 4 of the *BMP4* gene in hemochromatosis patients without known HH-related gene variants. However, the pathological mechanisms of BMP4 p.R269Q and p.H251Y need to be explicit.

Bone morphogenetic proteins (BMPs) are a subgroup with more than 20 members, which have been found to play important roles in regulating bone organ formation, embryonic development, neural development, and iron metabolism [15]. BMPs, including BMP6 and BMP2, can upregulate hepcidin expression levels by binding to BMP receptors and promoting the formation of the receptor-activated SMAD1/5/8-SMAD4 complex and its translocation to the nucleus [16]. Other data had also shown that human BMP4 and BMP9 can effectively upregulate hepcidin expression in primary mouse hepatocytes and the human hepatoma cell line HepG2 [17]. In recent years, mutations in BMP genes had been studied in hemochromatosis. Human heterozygous BMP6 deficiency causes a mild-to-moderate delayed iron overload phenotype, resulting in elevated serum ferritin levels and increased liver iron stores [18]. Another study analyzed the *BMP2* gene in 205 unrelated non-*HFE* hemochromatosis patients and found that the rs235768 (c.570A > T, p. Arg190ser) single nucleotide polymorphism (SNP) in the propeptide region of the *BMP2* gene was associated with iron overload [19]. However, the role of BMP4 and BMP4 variants in hemochromatosis pathogenesis remains unclear.

Our group had discovered the *BMP4* p.R269Q variant in one patient with unexplained primary iron overload through whole exome sequencing [11]. However, the pathogenicity of the *BMP4* p.R269Q variant in hemochromatosis remains unknown. In the present study, Sanger sequencing for the *BMP4* gene was conducted in patients with primary iron overload or secondary iron load, and we identified the *BMP4* p. H251Y variant in a patient with secondary iron overload. Furthermore, we found that the BMP4 p.H251Y and BMP4 p.R269Q variants can downregulate hepcidin expression levels by inhibiting the BMP/SMAD pathway. Therefore, these variants are potentially new pathogenic factors in hemochromatosis.

## Materials and methods

### Patients

A total of 54 patients with primary iron overload and 148 patients with secondary iron overload enrolled from the China Registry of Genetic/Metabolic Liver Diseases (CR-GMLD, Clinicaltrials.gov: NCT03131427) were recruited to screen for genetic analysis of variants in the *BMP4* gene. This study was conducted in accordance with the Clinical Research Ethics Committee of Beijing Friendship Hospital, Capital Medical University (No. 2016-P2-061–01). Written informed consent was obtained from all patients.

The clinical diagnosis of primary iron overload was based on the American Association for the Study of Liver Diseases 2011 practice guidelines on hemochromatosis [20]. The inclusion criteria are: (1) biochemical evidence showed that transferrin saturation (TS) ≥ 45% and/or elevated ferritin (> 200 ng/mL in premenopausal women or > 300 ng/mL in men and postmenopausal women); (2) it can be verified that iron overload in the liver and/or spleen on MRI (magnetic resonance imaging) of the liver or liver histology. The exclusion criteria (excluded causes of secondary iron overload) are: (1) chronic hepatitis B or C, alcoholic or other chronic liver disease; (2) parenteral iron overload; (3) iron-overloading anemia.

## Sanger sequencing for gene variants

Genomic DNA was extracted from whole blood using a QIAamp DNA blood Mini kit (Qiagen, Valencia, CA, USA) and then quantified using a NanoDrop 2000 spectrophotometer (Thermo Fisher Scientific, Waltham, MA, USA). The two protein-coding exons and the associated boundary regions were PCR-amplified using primers designed with Primer6. The primer sequences are listed in Supplementary Data-Table 1. PCR amplification was performed in a PCR cycler (Applied Biosystems; Thermo Fisher Scientific). The PCR products were sequenced in the forward and reverse orientations with an automated ABI 3730 DNA sequencer (Applied Biosystems). Three predictors, Polyphen-2 (http://genetics.bwh.harvard.edu), PROVEAN (http://provean.jcvi.org/index.php) and Mutation taster (http://www.mutationtaster.org/) were used to predict the functional consequence of the identified variants.

## Plasmids and RNA interference

The genes of *BMP4*, *BMP4* p.H251Y, and *BMP4* p.R269Q were amplified by polymerase chain reaction, and the coding sequence was cloned into the PCDH-CMV-MCS-EF1-copGFP-T2A-Puro vector to construct the wild-type BMP4 and BMP4 mutant plasmids. Transient transfection was performed with X-tremeGENE HP DNA transfection Reagent (Roche, Basel, Switzerland) according to the manufacturer’s instructions. BMP4 knockdown was performed using BMP4 siRNAs (ID SIGS0003737-1) (RiboBio, Guangzhou, China). Lipofectamine RNAiMAX reagent (Thermo, USA) was used for small interfering RNA transfection.

## Cell culture

The cell lines Huh7 and HepG2 were cultured respectively in Dulbecco’s Modified Eagle’s medium (Gibco, USA) and Minimum Essential Medium (Gibco, USA) supplemented with 10% FBS (Gibco, USA) and incubated at 37 °C with 5% CO2. The cell lines were obtained from the Cell Resource Center of the Chinese Academy of Medical Science (Beijing, China).

## RNA extraction and quantitative real-time PCR (qRT-PCR) analyses

TRIzol reagent (Invitrogen; Thermo Fisher Scientific) was used to extract total RNA from samples. The RNA was reverse transcribed into cDNA with a PrimeScript RT kit (Takara, Japan) according to the manufacturer’s protocols. Next, qRT-PCR was performed using a SYBR Green Real-Time PCR kit (Thermo Fisher Scientific) on the ABI PRISM 7300 RT-PCR system (Applied Biosystems). All reactions were run in triplicate and normalized to *GAPDH* mRNA levels using the 2^−ΔΔCt^ method. The primers were synthesized by Tianyi Huiyuan Biotechnology (Beijing, China).

The qRT-PCR primer sequences are as follows: *BMP4*: (forward) 5’-CTCCAAGAATGGAGGCTGTAGGAA-3’ and (reverse) 5’-CCTATGAGATGGAGCAGGCAAGA-3’; *HAMP*: (forward) 5’-TTTTCCCACAACAGACGGGA-3’ and (reverse) 5’-CTCCTTCGCCTCTGGAACAT-3’; *GAPDH*: (forward) 5’-CCTGCCAAGTATGATGACATCAAGA-3’ and (reverse) 5’-GTAGCCCAGGATGCCCTTTAGT-3’.

## Enzyme-linked immunosorbent assay (ELISA)

Cells were cultured for 48 h after transfection with small interfering RNAs (siRNAs) or plasmids, then the supernatant was collected and centrifuged to remove cell debris and impurities. Hepcidin levels in the cell culture supernatant were evaluated using the human hepcidin detection kit (Cloud-Clone Corp, Shanghai, China). The BMP4 detection kit (MultiScience Biotech, Hangzhou, China) was used to examine the BMP4 protein concentration in the supernatant.

## Western blot analysis

Cells were lysed on ice with radioimmunoprecipitation assay (RIPA) lysis buffer containing phosphatase and protease inhibitors (Roche, Basel, Switzerland) and collected. The protein concentration was determined by BCA assay (Yeasen Biotechnology, Shanghai, China), then denatured cell lysate samples (25 μg) with loading buffer were separated in 10% SDS-PAGE gels and transferred onto PVDF membranes (GE Healthcare, USA). After blocking with 5% fat-free milk at room temperature for 1 h, the membranes were incubated with specific primary antibodies. The following antibodies were used in the current study: anti-BMP4 (Abcam, Britain), anti-pSMAD1/5 (Thermo, USA), anti-tSMAD1 (Cell signaling technology, USA), anti-BMPR1A (ABclonal, Wuhan, China) and anti-GAPDH (Yeasen Biotechnology, Shanghai, China). After incubating with goat anti-rabbit and goat anti-mouse secondary antibodies (Zhongshan Golden Bridge, Beijing, China) for 1 h, the blots were visualized with ECL reagents (Yeasen Biotechnology, Shanghai, China) and quantified using Image J software (Millipore, USA).

## Statistical analysis

All data were presented as mean ± standard deviation (SD) from three independent experiments. All statistical analyses were performed by GraphPad Prism version 9 (GraphPad Software Inc, USA). The differences among groups of results were assessed by Student’s *t*-test. *p* < 0.05 was considered statistically significant.

## Results

### Clinical profiles of the HH patient with the BMP4 c.806G > A variant and secondary *iron* overload patient with the BMP4 c.751C > T variant

**Fig. 1:**
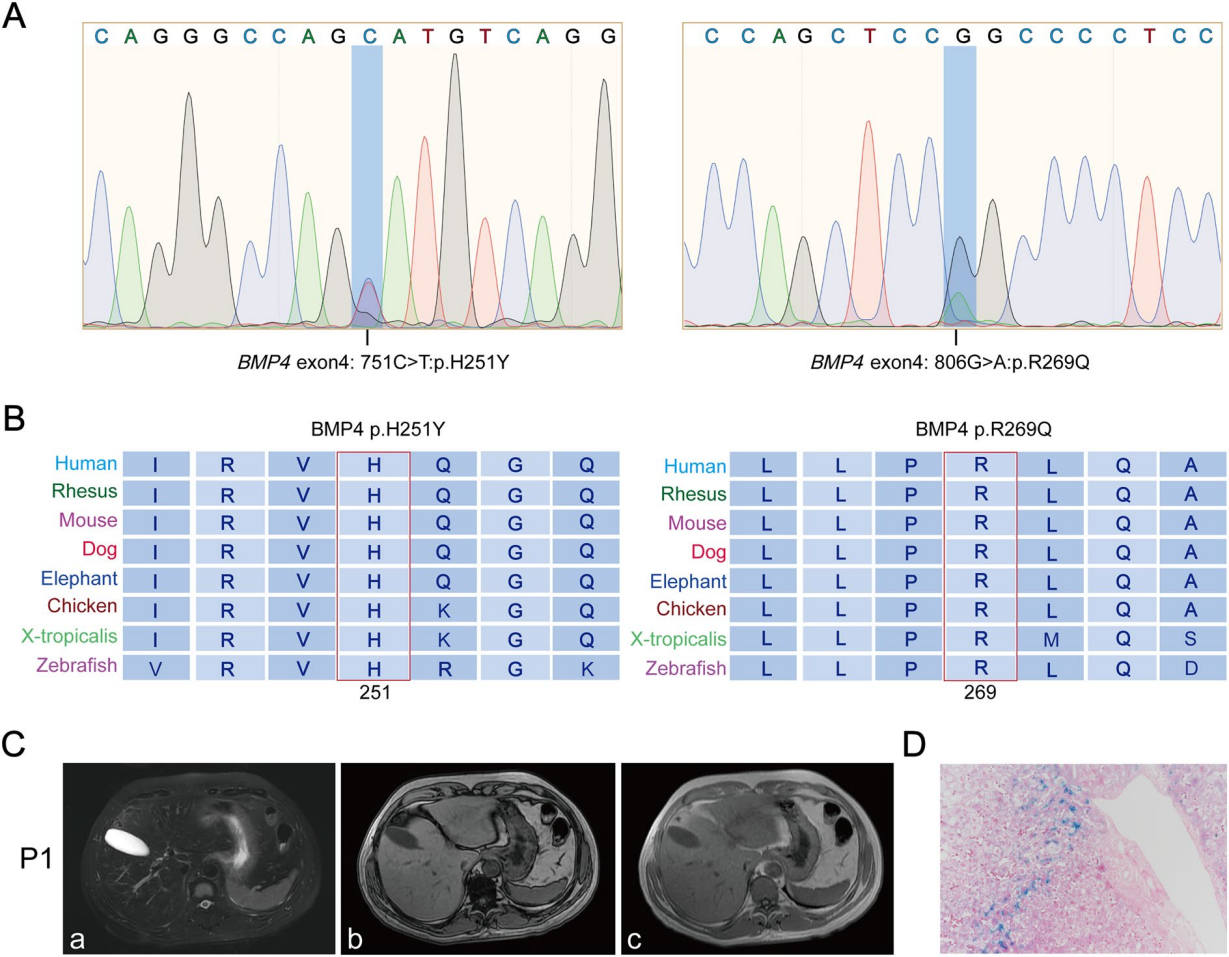
Analyses of two patients carrying *BMP4* p.H251Y and p.R269Q variants and their clinical characteristics. **A**. Sequencing peak chart of the heterozygous *BMP4* p.H251Y and p.R269Q variants. **B**. Alignment of amino acid sequences of human, rhesus, mouse, dog, elephant, chicken, x-tropicalis and zebrafish with regions flanking p.H251Y and p.R269Q variant sites. The positions of the BMP4 variants were indicated by the red box. Alignment was performed by http://genome.ucsc.edu/. **C**. MRI of the patient who carried *BMP4* p.R251Y. The signal intensity of the liver and spleen decreased on the T2 weighted image, indicating iron overload (a). On the T1 GRE dual-echo images (b and c), the signal intensity of the liver and spleen was lower on the in-phase image (c) than on the out-phase image (b), also indicating iron accumulation. **D**. A liver biopsy of the patient with hemochromatosis who carried *BMP4* p.H251Y showed that iron (Prussian blue staining particles) deposited largely in hepatocytes around portal areas and scarcely seen in Kupffer cells

In our previous study, whole exome sequencing was used to identify a novel heterozygous allelic variant c.806G > A in exon 4 of the *BMP4* gene. Then we performed Sanger sequencing for *BMP4* in 54 primary iron overload patients and 148 patients with secondary iron overload. Sanger sequencing showed another heterozygous allelic variant c.751C > T in *BMP4* in one of the 148 patients with secondary iron overload related to alcoholism. In contrast, the two *BMP4* variants were not found in 100 individuals from a Chinese general population. The c.751C > T variant involves a histidine to tyrosine change at position 251 (p.H251Y), while the c.806G > A variant has an arginine to glutamine change at position 269 (p.R269Q) (Fig. 1A).

In addition, sequencing for common iron regulatory genes (*HFE*, *HJV*, *HAMP*, *TFR2*, and *SLC40A1*) in HH indicated that the patient (P1) with the *BMP4* p.H251Y heterozygous genotype also had *SCL40A1* IVS1-8 variant and the patient (P2) with the *BMP4* p.R269Q heterozygous genotype also had *HJV* p.E3D variant (Supplementary Data-Table 2). Software prediction demonstrated that the two *BMP4* variants are likely damaging (Supplementary Data-Table 3). Sequence comparisons showed that amino acids 251 and 269 of BMP4 are conserved among different species (Fig. 1B). The clinical features of patients P1 and P2 with *BMP4* variants were shown in Supplementary Data-Table 2, with elevated levels of ferritin and transferrin in both patients. In addition, the son of HH patient P2 also had the *BMP4* R269Q variant, showing normal ferritin levels but increased transferrin saturation (TS) at 46%. The family segregation analysis of P2 patient was demonstrated in Supplementary Data-Figure S1. Liver iron deposition was confirmed in patient P1 by liver MRI examination and Perl's staining of liver biopsy (Fig. 1C and D).

## Hepcidin expression levels were decreased in BMP4-knockdown cell lines through the SMAD pathway

**Fig. 2:**
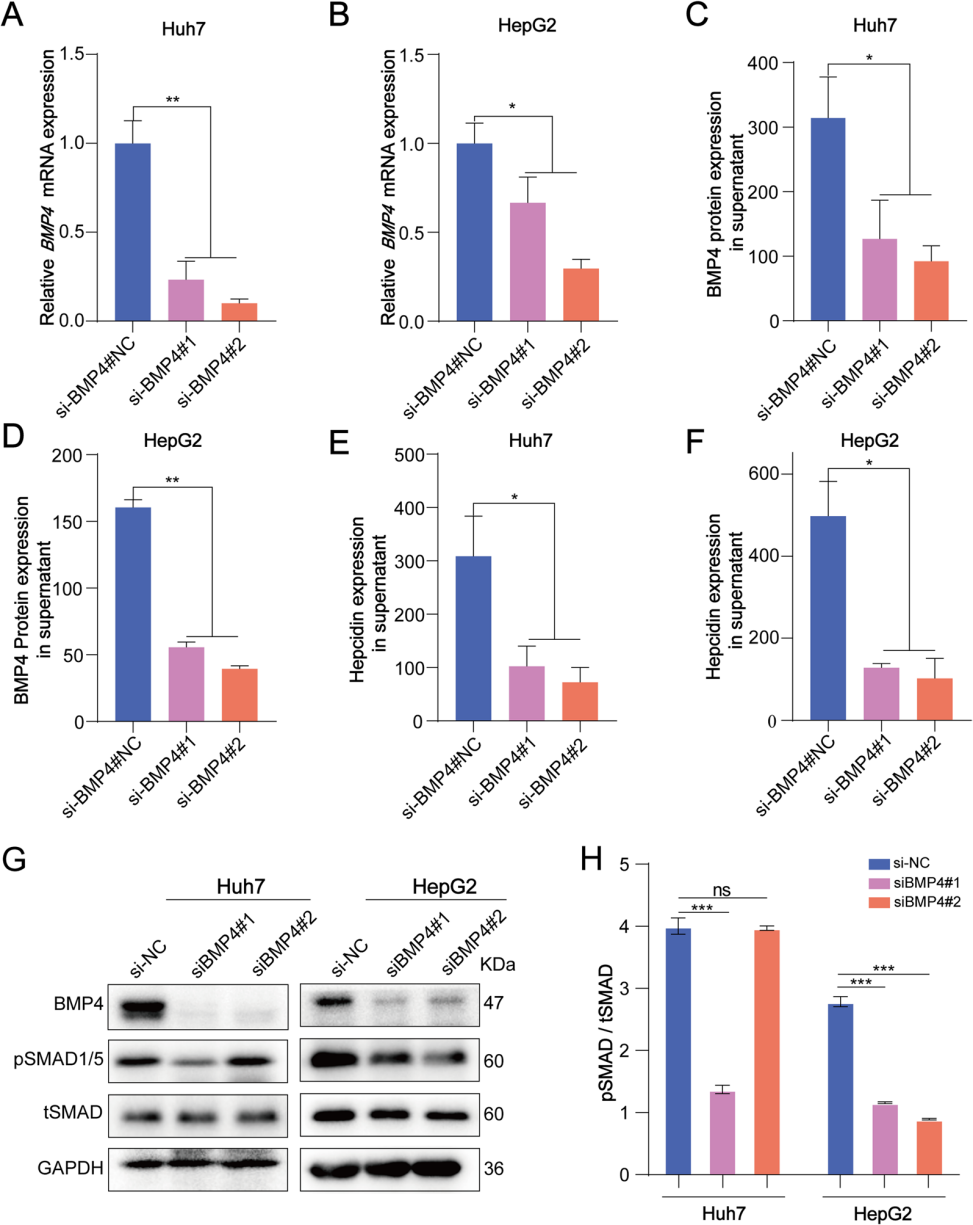
BMP4 knockdown decreased hepcidin expression by the SAMD pathway. **A** and **B**. BMP4 siRNAs were transducted into Huh7 and HepG2 cells. q-RT-PCR indicated that *BMP4* was knockdown. **C** and **D**. ELISA were used to analyze the concentration of BMP4 in the supernatant. **E** and **F**. ELISA for hepcidin revealed that BMP4 knockdown downregulates the Hepcidin expression level. **G** and **H**. BMP4 knockdown downregulated pSMAD1/5 pathway. The histogram showed relative analysis

Hepcidin is the dominating hormone in iron-regulatory homeostasis and BMPs have been found to play an important role in hepcidin regulation [21]. Therefore, we investigated if BMP4 is involved in regulating hepcidin expression. Two independent siRNAs were used to downregulate BMP4 expression in Huh7 and HepH2 cell lines, and then qRT-PCR and western blot analyses were used to examine their efficiency (Fig. 2A, B, and G). In both cell lines, BMP4 knockdown resulted in decreased BMP4 supernatant concentrations (Fig. 2C and D) and markedly reduced hepcidin levels (Fig. 2E and F). Additionally, western blot analysis showed that the expression of pSMAD1/5 was significantly downregulated in Huh7 cells transfected with si-BMP4#1 and in HepG2 cells transfected with both two BMP4-siRNAs (Fig. 2G and H). These data indicated that BMP4 knockdown downregulated hepcidin levels via the SMAD pathway.

## The BMP4 p.H251Y and BMP4 p.R269Q variants can downregulate hepcidin expression levels via inhibiting the BMP/SMAD pathway

**Fig. 3:**
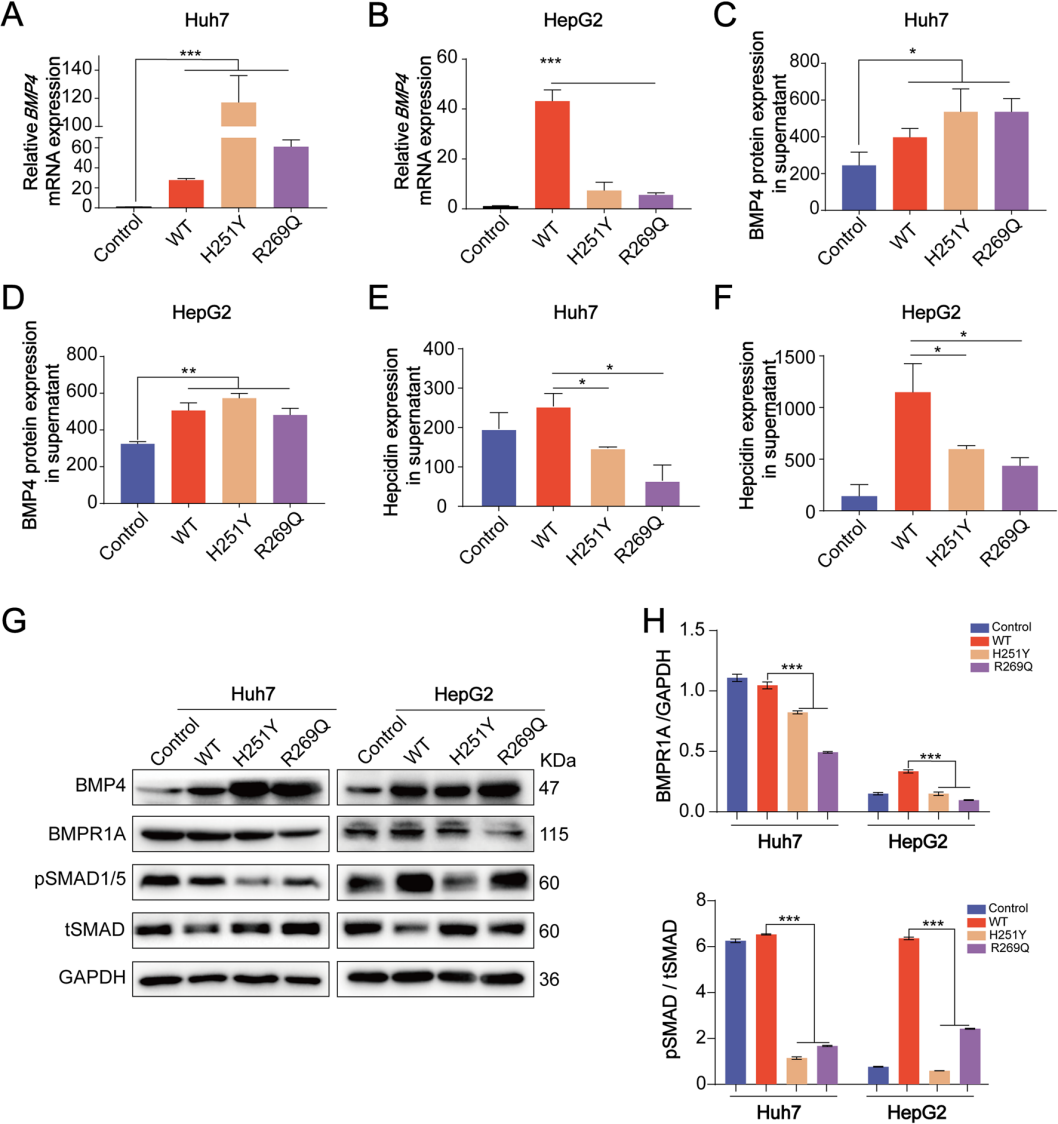
BMP4 variants down-regulated hepcidin level via inhibiting the BMP/SMAD pathway. **A** and **B**. q-RT-PCR showed *BMP4* expression in BMP4 p.H251Y and BMP4 p.R269Q variant cells. **C** and **D**. ELISA was used to analyze the concentration of BMP4 in the supernatant. **E** and **F**. ELISA showed that BMP4 p.H251Y and BMP4 p.R269Q variants led to decreased hepcidin expression compared with cells transfected with the wild-type BMP4 vector. **G** and **H**. Western blotting showed the BMP4 p.H251Y and BMP4 p.R269Q variants down-regulate the expression of BMPR1A and pSMAD1/5. The histogram represented a relatively quantitative expression analysis

To evaluate the effects of the BMP4 p.H251Y and BMP4 p.R269Q variants on hepcidin levels, we transfected Huh7 and HepG2 cells with the wild-type BMP4 and BMP4 variant vectors, then evaluated BMP4 expression levels using qRT-PCR and western blot analyses (Fig. 3A, B, and G). ELISA results showed that cells transfected with the wild-type BMP4 vector and BMP4 variants all elevated BMP4 supernatant concentrations (Fig. 3C and D). Transfection of the BMP4 p.H251Y and BMP4 p.R269Q variants led to lower hepcidin levels compared with the wild-type BMP4 vector (Fig. 3E and F).

Additional studies were performed to further elucidate the molecular mechanism of BMP4 variants on hepcidin regulation. Western Blot analysis revealed that BMPR1A expression and SMAD1/5 phosphorylation levels were downregulated in the cells transfected with the BMP4 p.H251Y or BMP4 p.R269Q vector compared with the wild-type BMP4 vector (Fig. 3G and H). Taken together, these results suggested that the BMP4 p.H251Y and BMP4 p.R269Q variants can downregulate hepcidin levels through the inhibition of the BMP/SMAD pathway.

## Discussion

In this study, we investigated the clinicopathological manifestation and functional analyses of the p.H251Y and p.R269Q variants in exon 4 of the *BMP4* gene, which have not been previously characterized. We found one patient carrying the *BMP4* p.R269Q variant among 54 primary iron overload patients and one patient harboring the *BMP4* p.H251Y variant among 148 patients with secondary hemochromatosis. Our experimental results suggested that the BMP4 p.H251Y and p.R269Q variants decreased hepcidin levels via the downregulation of the BMP/SMAD signaling pathway, indicating that the BMP4 p.H251Y and p.R269Q variants may be novel pathogenic factors of hemochromatosis.

In Caucasian HH patients, *HFE* p.C282Y is the most common mutation amounting to 1/220–250, with a mutation rate of 85%-90% of cases [22]. Unlike Caucasians, mutations in Chinese HH patients are predominantly non-*HFE* gene mutations. Compound heterozygous mutations of HJV and combined heterozygous mutations in BMP/SMAD pathway-related genes may be the major causative mutations of HH in China [11]. We previously had detected one patient carrying the *BMP4* p.R269Q variant by Exome sequencing [11], and then we found one carrying the *BMP4* p.H251Y variant by Sanger sequencing on exon 4 of the *BMP4* gene. Two variants were not found in 100 healthy general population. Therefore, it suggested that these two *BMP4* variants may be hemochromatosis pathogenic.

Previous studies had shown that holo-transferrin can regulate *HAMP* mRNA levels in fresh isolated murine primary hepatocytes through the hemoglobin/BMP2/4-dependent pathway, as well as that BMP4 can induce hepcidin transcription independently of HJV in HepG2 cells [23, 24]. Here, we measured the hepcidin levels in BMP4 knockdown cells to explore the functional role of BMP4 in hepcidin and iron homeostasis regulatory mechanisms. We observed decreased hepcidin levels in the Huh7 and HepG2 cell supernatant after BMP4 knockdown. The BMP-SMAD signaling pathway is known to be central to regulating hepcidin transcription [16]. Furthermore, BMP4 can also inhibit fibroblast activation and differentiation by activating SMAD1/5/9 signaling and suppressing SMAD2/3 signaling [25]. Our study demonstrated that BMP4 knockdown could downregulate pSMAD1/5 expression levels, suggesting that BMP4 has a potential functional role in hepcidin regulatory mechanisms through the SMAD pathway.

Currently, the BMP4 gene variants and pathogenesis in hemochromatosis are rarely studied. Mile et al. reported a significant association between serum ferritin level and *BMP2* rs235756 SNP in patients with *HFE*-associated hemochromatosis, and there was a small additive effect of *BMP4* SNP rs4901474 on the hemochromatosis penetrance [26]. To investigate if the BMP4 p.H251Y and p.R269Q variants could downregulate hepcidin expression levels and explore any subsequent effects on iron metabolism-related signaling, we examined hepcidin concentrations in the supernatant of cells transfected with the wild-type BMP4 and BMP4 variant vectors. Compared with cells transfected with the wild-type BMP4 vector, cells transfected with the BMP4 p.H251Y or p.R269Q variant had significantly decreased hepcidin levels in the supernatant. Because we previously demonstrated that BMP4 knockdown decreased hepcidin levels by inhibiting the SMAD pathway, we explored the mechanism by which BMP4 variants can regulate iron metabolism-related pathways. When the plasma or hepatocyte iron concentration increases, BMP/SMAD signaling pathways will be activated and then bind to type I and type II receptors to induce hepcidin expression [22]. BMP2 and BMP4 have been reported to bind preferentially to BMPR1A and BMPR1B and then to recruit type II receptors [15, 27]. Recent studies also indicated that BMP4-BMPR1A signaling regulates several biological functions including cell proliferation and migration and insulin secretion [28, 29]. Our data demonstrated that BMPR1A and pSMAD1/5 expression levels were downregulated following transfection of the BMP4 p.H251Y or BMP4 p.R269Q variant vectors compared with wild-type BMP4 vector transfection. These results suggested that the BMP4 p.H251Y and BMP4 p.R269Q variants downregulated hepcidin levels via the inhibition of the BMP/SMAD signaling pathway.

In addition, although the patient with the *BMP4* p.H251Y variant was diagnosed as secondary iron overload in the clinical context, we speculate that the presence of this *BMP4* variant may increase the risk of developing iron overload with the condition of alcoholism.

However, this study does have limitations. First, hemochromatosis is a rare disease in China, so the number of cases we were able to collect was limited. Further study with more cases is needed to make a more robust conclusion. Second, we did not develop a mouse model to verify the effects of these BMP4 variants on the BMP/SMAD pathway and iron metabolism regulation by hepcidin in vivo. Additional experimental data are required to support our present conclusions in future work. The cohort of Chinese patients with primary iron overload should be expanded in the future to analyze the role of the BMP4 p.H251Y and p.R269Q variants in hemochromatosis and obtain a more comprehensive understanding of the relevant pathological mechanisms.

## Conclusion

Our results indicated that the *BMP4* p.H251Y and *BMP4* p.R269Q variants in exon 4 of the *BMP4* gene can downregulate hepcidin production by inhibiting the BMP/SMAD axis, suggesting their potential role in the pathogenesis of hemochromatosis.

## Supplementary Information

Additional file 1.

Additional file 2.

## Data Availability

All data generated or analyzed during this study are included in this published article (and its supplementary information files).

## Abbreviations

*HH*: Hereditary hemochromatosis
*HFE*: Homeostatic iron regulator
*HJV*: Hemojuvelin
*HAMP*: Hepcidin antimicrobial peptide
*TFR2*: Transferrin receptor 2
*SLC40A1*: Solute carrier family 40 member 1
*TMPRSS6*: Transmembrane serine protease 6
*DENND3*: DENN domain containing 3
*SUGP2*: SURP and G-patch domain containing 2
*UBE2O*: Ubiquitin-conjugating enzyme E2 O
*PCSK7*: Proprotein convertase subtilisin/kexin type 7
*TS*: Transferrin saturation
*SNP*: Single nucleotide polymorphism
*BMP*: Bone morphogenetic protein
*SMAD*: Small mothers against decapentaplegic
*BMP4*: Bone morphogenetic protein 4
