# Supplementary material for: Recurrent BMP4 variants in exon 4 cause non-HFE-associated hemochromatosis via the BMP/SMAD signaling pathway
Source: Orphanet J Rare Dis. 2024 Nov 19;19:429. doi: 10.1186/s13023-024-03439-9 (PMC11575201; doi:10.1186/s13023-024-03439-9)
Supplement: Supplementary file 1 — Additional file 1. [file 13023_2024_3439_MOESM1_ESM.docx]

Table 1. PCR primers for Sanger sequencing of *BMP4*

| Exons | Forward | Reserve |
| --- | --- | --- |
| BMP4-3 | GGTGGTGTGAGGGAGAAGAC | CTGGACTGGGGCTTTGATGT |
| BMP4-4-1 | TGTTAGCTGCCCCACTTATCTG | TGGCATGGTTGGTTGAGTTGA |
| BMP4-4-2 | CGATCGTTACCTCAAGGGAGT | GCACGTAAAGTCATAAATAAGGTCA |

|  | **Age** | **Gender** | **SF (ng/ml)** | **TS (%)** | **Iron deposition on liver biopsy** | **Iron overload on MRI** | **End-organ manifestations** | **Mutation in known HH-related genes** |
| --- | --- | --- | --- | --- | --- | --- | --- | --- |
| P1 | 61 | M | 1151 | 81.8 | Mild in periportal hepatocytes | Liver and spleen | None | *SLC40A1* IVS1-8 |
| P2 | 53 | F | 1402 | 49.0 | Predominant in hepatocytes | Liver and spleen | Lethargy, jaundice, abnormal liver function test | *HJV p.E3D* |

Table 2. Clinical characteristics of patients with hemochromatosis

Table 3. In silico analysis of the non-synonymous variants in the iron metabolism-related genes identified in HH with single *DENND3* p.L708V

| Gene | Amino  Acid  change | Base  change | Polyphen-2 | | PROVEAN | | Mutation Taster | | |
| --- | --- | --- | --- | --- | --- | --- | --- | --- | --- |
|  |  |  | Prediction | Score | Prediction | Score | Prediction | Score  (Prob) | Score  (AA change) |
| BMP4 | p.H251HY | c.751C>CT | Probably Damaging | 0.996 | Deleterious | -5.015 | Disease causing | 1.000 | 83 |
| BMP4 | p.R269RQ | c.806G>GA | Probably Damaging | 0.995 | Deleterious | -3.114 | Disease causing | 1.000 | 43 |


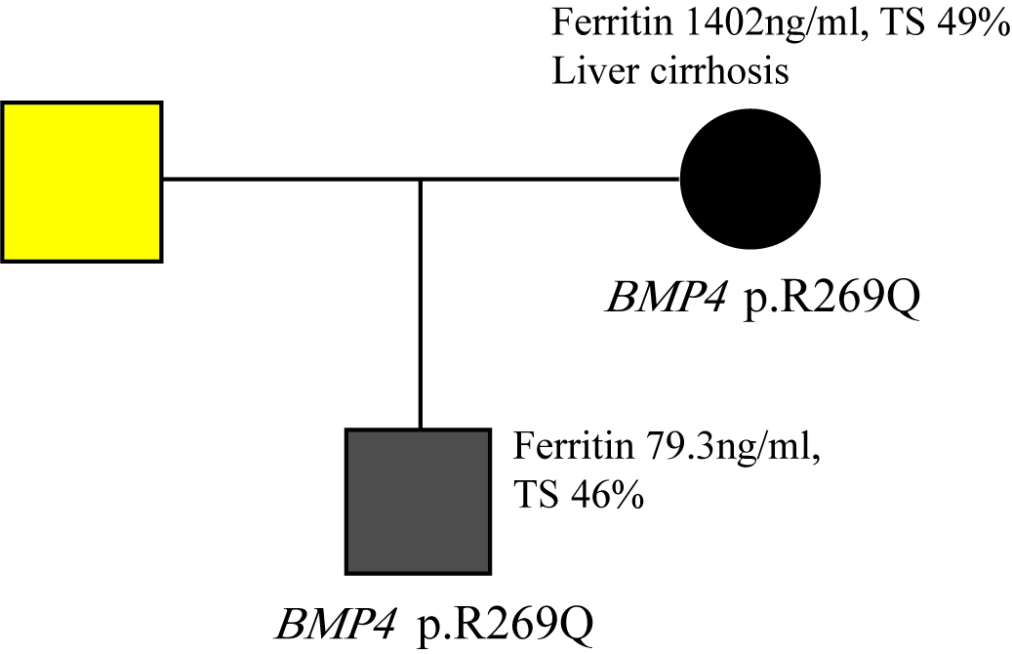


**Figure S1.The family segregation analysis of P2 patient showed that *BMP4* p.R269Q variant was associated with hemochromatosis.** The precursor who carried the *BMP4* p.R269Q is a woman with significant iron overload and liver cirrhosis. The patient’s son was genetically tested to carry the *BMP4* p.R269Q variant, showing normal ferritin levels but increased transferrin saturation (TS) at 46%.
