# Supplementary figures and images for: Recurrent BMP4 variants in exon 4 cause non-HFE-associated hemochromatosis via the BMP/SMAD signaling pathway
Source: Orphanet J Rare Dis. 2024 Nov 19;19:429. doi: 10.1186/s13023-024-03439-9 (PMC11575201; doi:10.1186/s13023-024-03439-9)

Figure2G-western blot

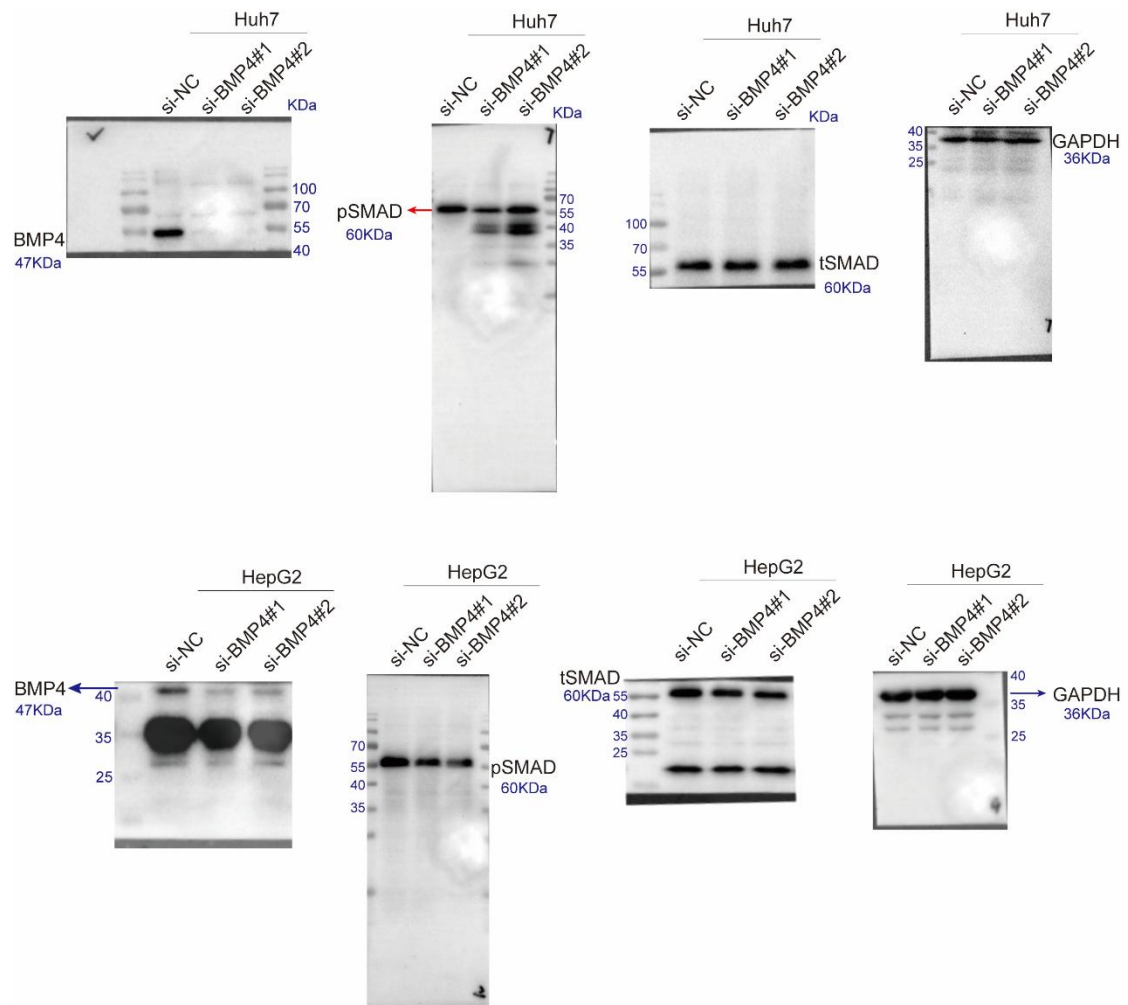

Figure 3G-western blot

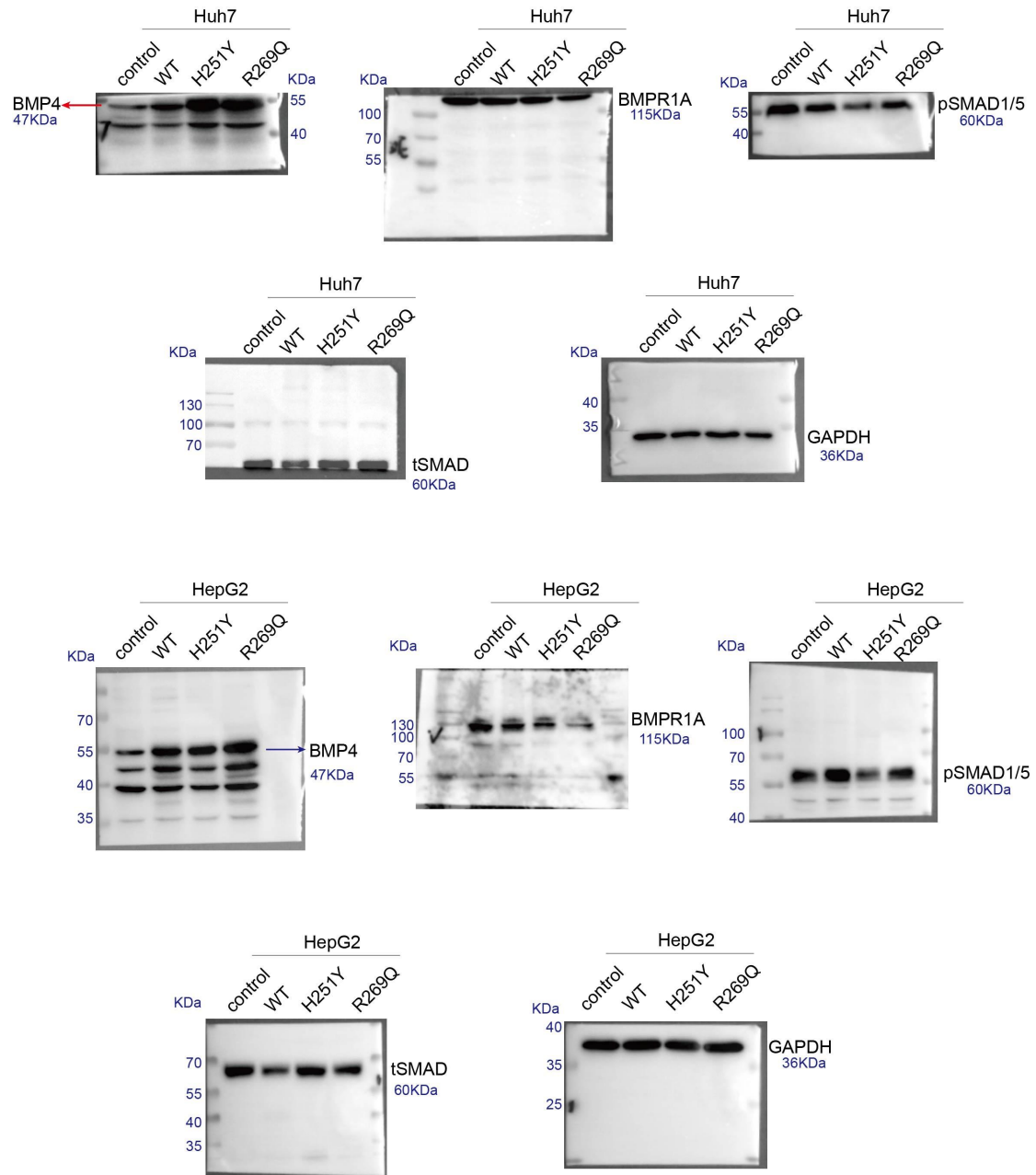

Supplement: Supplementary file 2 — Additional file 2. [file 13023_2024_3439_MOESM2_ESM.pdf]
